# Inter-plasmid transfer of antibiotic resistance genes accelerates antibiotic resistance in bacterial pathogens

**DOI:** 10.1093/ismejo/wrad032

**Published:** 2024-01-10

**Authors:** Xiaolong Wang, Hanhui Zhang, Shenbo Yu, Donghang Li, Michael R Gillings, Hongqiang Ren, Daqing Mao, Jianhua Guo, Yi Luo

**Affiliations:** College of Environmental Science and Engineering, Ministry of Education Key Laboratory of Pollution Processes and Environmental Criteria, Nankai University, Tianjin 300071, China; State Key Laboratory of Pollution Control and Resource Reuse, School of the Environment, Nanjing University, Nanjing 210023, China; State Key Laboratory of Pollution Control and Resource Reuse, School of the Environment, Nanjing University, Nanjing 210023, China; School of Medicine, Nankai University, Tianjin 300071, China; ARC Centre of Excellence in Synthetic Biology, Faculty of Science and Engineering, Macquarie University, Sydney, New South Wales 2109, Australia; State Key Laboratory of Pollution Control and Resource Reuse, School of the Environment, Nanjing University, Nanjing 210023, China; School of Medicine, Nankai University, Tianjin 300071, China; Australian Centre for Water and Environmental Biotechnology, The University of Queensland, Brisbane, Queensland 4072, Australia; College of Environmental Science and Engineering, Ministry of Education Key Laboratory of Pollution Processes and Environmental Criteria, Nankai University, Tianjin 300071, China; State Key Laboratory of Pollution Control and Resource Reuse, School of the Environment, Nanjing University, Nanjing 210023, China

**Keywords:** antimicrobial resistance, plasmids, antibiotic resistance genes, ESKAPE pathogens, horizontal gene transfer, bioinformatics

## Abstract

Antimicrobial resistance is a major threat for public health. Plasmids play a critical role in the spread of antimicrobial resistance via horizontal gene transfer between bacterial species. However, it remains unclear how plasmids originally recruit and assemble various antibiotic resistance genes (ARGs). Here, we track ARG recruitment and assembly in clinically relevant plasmids by combining a systematic analysis of 2420 complete plasmid genomes and experimental validation. Results showed that ARG transfer across plasmids is prevalent, and 87% ARGs were observed to potentially transfer among various plasmids among 8229 plasmid-borne ARGs. Interestingly, recruitment and assembly of ARGs occur mostly among compatible plasmids within the same bacterial cell, with over 88% of ARG transfers occurring between compatible plasmids. Integron and insertion sequences drive the ongoing ARG acquisition by plasmids, especially in which IS26 facilitates 63.1% of ARG transfer events among plasmids. *In vitro* experiment validated the important role of IS26 involved in transferring gentamicin resistance gene *aacC1* between compatible plasmids. Network analysis showed four beta-lactam genes (*bla_TEM-1_*, *bla_NDM-4_*, *bla_KPC-2_*, and *bla_SHV-1_*) shuffling among 1029 plasmids and 45 clinical pathogens, suggesting that clinically alarming ARGs transferred accelerate the propagation of antibiotic resistance in clinical pathogens. ARGs in plasmids are also able to transmit across clinical and environmental boundaries, in terms of the high-sequence similarities of plasmid-borne ARGs between clinical and environmental plasmids. This study demonstrated that inter-plasmid ARG transfer is a universal mechanism for plasmid to recruit various ARGs, thus advancing our understanding of the emergence of multidrug-resistant plasmids.

## Introduction

Antimicrobial resistance is a global challenge to public health, claiming over 1.3 million lives in 2019 [1]. Plasmids are capable of moving between bacteria and are recognized as being important vehicles that transfer antibiotic resistance genes (ARGs) between bacterial species [2, 3]. The emergence of antimicrobial resistance in clinical pathogens is frequently associated with plasmids [4-6]. For instance, the plasmid-borne carbapenem resistance genes *bla_KPC_* [7] and *bla_OXA-48_* [8] and the colistin resistance gene *mcr* [9] are spreading in clinical pathogens under antibiotics selective pressure. Crucially, some of these plasmid-bearing bacteria are multidrug-resistant pathogens, thus becoming “superbugs” that disseminate uncontrollably in clinical settings. These include carbapenem-resistant *Klebsiella pneumoniae* [10], *Acinetobacter baumannii* [11], *Pseudomonas aeruginosa* [12], and *Enterobacter* spp. [13], and colistin-resistant *Enterobacteriaceae* [9].

It is well acknowledged that plasmids are mobile vectors to carry ARGs in clinical pathogens, however, little is known about how plasmids originally acquire ARGs. Previous studies mainly focused on conjugative plasmids and suggested that bacterial hosts acquire antibiotic resistance via incorporation of these plasmids. In the presence of antibiotic stress, these plasmids are beneficial for host bacteria since they act as flexible storage elements to acquire and rearrange exogenous genetic traits from different genetic contexts.

Notably, some antibiotic-resistant bacteria can carry multiple plasmids [14, 15], which provides an opportunity for plasmids to exchange ARGs. Multiple plasmids that coexist in the same bacterial cell are defined as compatible plasmids, while plasmids with inability to coexist in the same bacterial host are defined as incompatible [16]. Previous studies reported that high-sequence similarity regions are frequently observed in otherwise unrelated plasmid genomes, indicating that there are other mobile genetic elements (MGEs) moving between plasmids [17]. Based on these observations, it is hypothesized that plasmids could exchange MGEs and their accessory sequences, thus acquiring various ARGs. Previous study reported that plasmids can acquire ARGs from phylogenetically distant chromosomes [18], this being one of the routes for plasmids to acquire ARGs. However, it is still unknown how plasmid originally recruit ARGs from coexistence plasmids.

The objective of this study was to decipher whether and how ARG transfer occurs between plasmids in clinical pathogens. To this end, 2420 clinically relevant and 882 environmentally relevant conjugative plasmids with complete sequences were collected from public databases. These plasmids were used to identify potentially recently transferred ARGs and then to determine the horizontal transfer of ARGs and associated MGEs across the plasmids by systematic genetic analysis in conjunction with experimental validation. Additionally, a multilevel network analysis was conducted to assess the effect of inter-plasmid ARG transfer on antibiotic resistance proliferation in clinical pathogens. Finally, comparisons of ARG nucleotide sequence similarity between plasmid vectors were conducted to assess the potential for ARG transfer between environmental and clinical plasmids. This is the first study to reveal a universal model of inter-plasmid ARG transfer among various clinical pathogens. The findings offer insights into how plasmids recruit diverse ARGs and will help to formulate strategies for mitigating the spread of antimicrobial resistance.

## Materials and methods

### Complete bacterial plasmid collection

In this study, 27 938 complete plasmids were first downloaded from the NCBI RefSeq database (ftp://ftp.ncbi.nlm.nih.gov/refseq/release/plasmid/) in November 2020 in GenBank and FASTA formats. Among 27 938 plasmids, 3302 conjugative plasmids were classified as clinical and environmental plasmids for subsequent plasmid classification and ARG identification. The annotation features including bacterial host and isolation source were automatically extracted from GenBank files using a local Python script. The Python script was uploaded in GitHub (https://github.com/loong91/Inter-plasmid-ARG-transfer/blob/main/genbank.py). For each plasmid GenBank file, the Python script enables to find the certain characteristics (e.g. “isolation source,” “organism,” and “collection data”) to extract the feature annotation and export the information to a table. According to “isolation source,” the plasmid isolated from human blood, urine, fecal swab, feces, or skins in hospitals were categorized as cliniclike plasmids. From the plasmid database, we cannot identify if the host bacteria of plasmid are pathogenic to humans; therefore, we used a loose definition of clinical plasmids and make more accurate to call cliniclike plasmids. Plasmids isolated from soil, wastewater, river, sediment, air, and other environmental niches were classified as environmental plasmids. When the information either “isolation source” or “organism” was missing, the category of plasmid was manually confirmed through searching literature. After the classification, we obtained 2420 clinical conjugative plasmids (Supplementary Table S1) and 882 environmental conjugative plasmids (Supplementary Table S2) for subsequent analyses.

### Plasmid classification

To retrieve conjugative plasmids from the plasmid database, plasmid classification was conducted based on plasmid mobility. All retrieved plasmids were classified into conjugative, mobilizable, and nonmobilizable plasmids according to the protein machinery for DNA transfer, including relaxase, Type IV coupling protein, and Type IV secretion system, as suggested by previous studies [19-21]. Conjugative plasmids were used for subsequent analyses. Then, the taxonomic classifier of plasmids, COPLA, was used to assign plasmids to taxonomic units with default parameters [22]. Pairwise ANI approach is not suitable for plasmid classification because there is no universal core of genes shared by plasmids, as well as MGEs often hop on plasmid genomes. Pairwise ANI for plasmids would cause unrelated plasmids displaying specific genome fragments with a high average nucleotide identity value. To improve the prevision and universality of plasmid classification, the COPLA approach combined average nucleotide identity approach and alignment fraction, which correct the bias caused by MGEs hopping in plasmid genomes. Results of plasmid taxonomic units are shown in Supplementary Table S1 and Supplementary Table S2. The key components for conjugative plasmids are shown in Supplementary Table S3.

### Identification of antibiotic resistance gene and metal resistance genes

To identify plasmid-borne ARGs, the open reading frames (ORFs) were firstly predicted by Prodigal (v2.6.3) with default parameters. All ORFs of plasmids were aligned to SARG database [23] to identify ARGs using BLASTp with parameters of E-value ≤1e-5, minimum similarity of 90% and query coverage >80%. ORFs of plasmids were aligned to the BacMet database (version 2.0), containing experimentally confirmed biocides, and metal resistance genes (MRGs) were used to identify MRGs. The MRGs results are presented in Supplementary Tables S4 and S5.

### Mobile genetic element identification

To identify MGEs associated with recently transferred ARGs, the integrons and insertion sequences (ISs) were then identified. Integrons carried by plasmids were identified using the Integron Visualization and Identification Pipeline (https://github.com/caozhichongchong/I-VIP) with default settings [24]. ISs were detected based on homology search against the ISFinder database using BLASTn (E-value ≤1e-10) [25] with similarity of 80% and coverage of 80%. To determine association between ISs and ARGs on plasmids, the adjacent sequences, i.e. 5 kb upstream and downstream of ARGs, were extracted for the identification of ISs. The MGEs identification are included in Supplementary Table S6.

### Determination of the potentially recently transferred antibiotic resistance genes and horizontal gene transfer frequency

**Figure 1:**
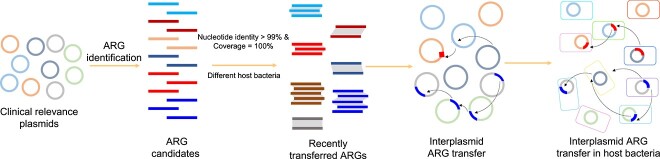
Workflow for identification of recently transferred ARGs in plasmids.

To identify potentially inter-plasmid ARG transfer, we adopted a widely used bioinformatic analysis approach to detect nearly identical full ARG sequence across plasmids [18, 26-30]. The occurrence of nearly identical full DNA sequences of ARGs between different species is assumed to be attributed to horizontal gene transfer, and operationally defined as recently transferred ARGs [18, 26], as an *ad hoc* approach to identify potential horizontal gene transfer (HGT) events between plasmids. In this study, potentially recently transferred ARGs were defined as those with perfect identity (more than 99% nucleotide identity and 100% coverage) in distinct plasmids in distinct host bacteria using BLASTn (E-value ≤10^−5^) [18]. To minimize the impact of plasmid transfer on the observed occurrence of inter-plasmid ARG transfer, the distinct plasmids were defined as those with a nucleotide identity of <80% and a coverage of <80% in pairwise alignment. According to the definition of recently transferred genes, transferred genes larger than 500 bp with >99% similarity are consistent with transfer events that occurred between 0 and 10 000 years [28]. Specifically, the candidate ARGs were extracted from the plasmid sequence, and the pairwise alignment of candidate ARGs was conducted. Based on these cut-off values, the recently transferred ARGs among clinical plasmids or among clinical and environmental plasmids were identified using the same approach. Then, HGT was counted as the number of between-bacterial host pairs (species level) sharing at least one HGT. To measure the frequency of HGT between two species, we then divided the HGT count by the total number of between-bacterial host pairs (species level). The schematic diagram for bioinformatic analyses in this study is illustrated in Fig. 1.

### Identification of ESKAPE pathogens harboring plasmid-borne antibiotic resistance genes

The acronym ESKAPE includes six nosocomial pathogens that exhibit multidrug resistance and virulence: *Enterococcus faecium*, *Staphylococcus aureus*, *K. pneumoniae*, *A. baumannii*, *P. aeruginosa*, and *Enterobacter* spp. ESKAPE pathogens are responsible for majority of nosocomial infections and are capable of “escaping” the biocidal action of antimicrobial agents. Now, ESKAPE pathogens [31] were designated “priority status” for which new antibiotics are urgently needed [32]. If a bacterial species for a plasmid was classified as an ESKAPE pathogen, then the plasmid was considered as the ESKAPE-harboring plasmid, and the plasmid-borne ARGs were considered as ESKAPE pathogens–harboring plasmid-borne ARGs.

### Network construction and analysis

To visualize the ARG transfer pattern across plasmids, the network was adopted to exhibit the physical linkage pattern of plasmids and ARGs. Gephi software was used for network visualization and manipulation, while the location of the ARGs and plasmids were determined using Fruchterman–Reingold as a layout algorithm [33]. In the network, nodes represented as plasmids or ARGs. If the ARG can transfer across plasmids, then there is one edge connecting the ARG and plasmids. The node size is proportional to the number of the edge. Similarly, the network was also used to visualize the linkage of plasmid and plasmid types, as well as plasmid-bearing ARGs and bacterial host.

### Construction of the pUC57-*lac*I^q^-IS*26*-P*lac*-gfp_mut3b_-Gm^+^-IS*26*

In order to experimentally confirm the contribution of IS26 on inter-plasmid ARG transfer, we constructed a plasmid carrying both IS26 and green fluorescent proteins (GFP), thus visualizing IS26-mediated ARG transfer across plasmids. Specifically, the sequence of IS*26* was synthesized by Tsingke Biotechnology Co., Ltd. and cloned into the region of EcoRI and HindIII site in the pUC57 plasmid, named pUC57-IS*26*. Then, the Plac-gfp_mut3b_, Gm^+^ (*aacC1*), and IS*26* were amplified from RP4-Plac-gfp_mut3b_ [34], pEX18Gm, and pUC57-IS*26*, separately. Three polymerase chain reaction (PCR) products (IS*26*, Plac-gfp_mut3b_, and aacC1) were linked together by overlap PCR. Then, the plasmid pUC57-IS*26*-Plac-gfp_mut3b_-Gm^+^-IS*26* was constructed by linking the fusion product (IS*26*-Plac-gfp_mut3b_-Gm^+^-IS*26*) and the plasmid pUC57. Subsequently, the *lac*I^q^ gene was amplified from the chromosome of *Escherichia coli (*E. coli*)* MG1655:lacI^q^-Plpp-mCherry [34], and *lac*I^q^ was inserted into the pUC57-IS*26*-Plac-gfp_mut3b_-Gm^+^-IS*26* plasmid. As a result, we obtained the plasmid pUC57-*lac*I^q^-IS*26*-Plac-gfp_mut3b_-Gm^+^-IS*26*, in which green fluorescence is inhibited by *lac*I^q^. The green fluorescence is visible once the gene cassette IS*26*-P*lac*-gfp_mut3b_-Gm^+^-IS*26* was transferred to other plasmids out of the bacteria, which did not carry *lac*I^q^ gene. The circular map of constructed plasmid was shown as Supplementary Fig. S1. The primers used in this experiment is shown in Supplementary Table S7. The full sequence of *lac*I^q^-IS*26*-Plac-gfp_mut3b_-Gm^+^-IS*26* is shown in Supplementary Table S8.

### Validation of IS*26*-mediated transposition across plasmids

The IncP plasmid pDTC28, carrying tetracycline resistance genes (*tetA*), was used to assess the IS*26*-mediated transfer across plasmids. The transposition process was first investigated via conjugation of pDTC28 plasmid into *E. coli* DH5α cells, which contain the nonconjugative plasmid pUC57-*lac*I^q^-IS*26*-P*lac*-gfp_mut3b_-Gm^+^-IS*26*. Transconjugants were selected for Amp^R^ Tet^R^ Gm^R^ colonies and incubated for 24 hours. Then, a further mating assay was conducted to assess whether IS*26* was able to mediate ARG transfer across plasmids. The *E.coli* DH5α harboring both pDTC28 and pUC57-*lac*I^q^-IS*26*-P*lac*-gfp_mut3b_-Gm^+^-IS*26* was selected as the donor, while plasmid-free *E. coli* K12 J53 (sodium azide resistance) was chosen as the recipient. The transconjugants were selected for Tet^R^, Gm^R^, sodium azide, and green fluorescent colonies, which could directly reflect the IS*26*-mediating ARG transfer. Apart from the plasmid pDTC28, RP4 was also selected to confirm the IS*26*-associated Gm^R^ transfer across plasmids.

### Statistical analysis

The network was visualized using Gephi version 0.9.2. The barplot, boxplot, and scatter plot were all plotted by R packages ggplot2. Correlation test was conducted by R packages. Statistical comparisons were done using nonparametric Wilcoxon tests. A *P*-value of <.05 was regarded as statistically significant.

## Results

### Inter-plasmid antibiotic resistance gene transfer accelerates plasmid recruiting clinically relevant antibiotic resistance genes

Plasmid serves as a genetic element on which ARGs are frequently recruited and assembled. To detect horizontal transfer of ARGs between plasmids, we retrieved 2420 clinically relevant conjugative plasmid sequences, spanning the years 1996 to 2020. We found the proportion of ARG-harboring plasmids accounted for 70.2% of the plasmid collection. In addition, these plasmids harbored diverse ARGs, including 8229 ARGs belonging to 12 antibiotic classes. Plasmid-borne beta-lactam ARGs exhibited the highest proportion (56.6%), followed by aminoglycoside (37.9%), and sulfonamide (32.5%) (Supplementary Fig. S2A). Among these plasmid-borne ARGs, we used ARG copy number per plasmid (Supplementary Fig. S2B) to define plasmid-borne ARG abundance. Results suggest that plasmid-borne ARGs resistance to aminoglycosides (average 2.33 ARG copies/plasmid) and beta-lactams (average 1.71 ARG copies/plasmid) were more abundant than other plasmid-borne ARGs (e.g. quinolone, macrolide–lincosamide–streptogramin (MLS) resistance genes).

**Figure 2:**
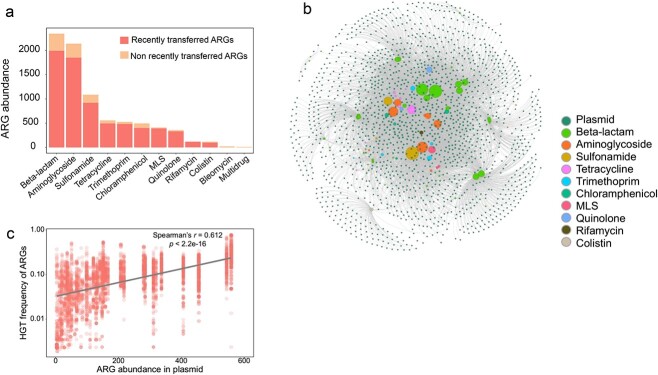
ARGs transfer across clinically relevant plasmids; (A) the number of ARGs in clinical plasmids; (B) the network connecting plasmids with recently transferred ARGs; (C) correlation of transfer frequency and number of ARGs in each plasmid.

Inter-plasmid ARG transfer could be an effective evolutionary strategy for bacteria to acquire ARGs and adapt to antibiotic stress. To identify potentially horizontal transfer of ARGs across plasmids, pairwise alignments of plasmid-borne ARGs were conducted. High nucleotide sequence identity (more than 99% identity) was considered to represent recently transferred ARGs [18, 26]. Accordingly, a total of 7129 ARGs were identified as being recently transferred across multiple plasmids. These recently transferred ARGs accounted for 86.6% of all ARGs harbored by plasmids (Fig. 2A), including those genes for resistance to beta-lactams, aminoglycosides, sulfonamide, tetracycline, trimethoprim, chloramphenicol, MLS, quinolone, rifampin, and colistin (Fig. 2A). These recently transferred ARGs belong to 77 ARG subtypes (Table 1). Among them, 37 ARG subtypes encoding resistance to beta-lactams and 11 ARG subtypes against aminoglycosides represented the two major classes of antibiotic resistance of concern in clinical settings (Table 1). Notably, ARGs toward last line antibiotics (e.g. *bla_NDM_*, *bla_CTX-M_*, and *mcr-1* genes against beta-lactam and colistin) could transfer between conjugative plasmids, highlighting the critical concern for plasmid-mediated antibiotic resistance in clinical pathogens.

**Table 1:** Recently transferred ARGs carried by plasmids.

ARG types	Recently transferred ARG subtypes
Aminoglycoside	*aac3-I*; *aac3-II*; *aac3-IV*; *aac6-I*; *aac6-II*; *aadA*; *ant2-I*; *aph3-I*; *aph4-I*; *aph6-I*; *rmtB*
Beta-lactam	*ampC*; *CMY-2*; *CMY-4*; *CTX-M*; *CTX-M-1*; *CTX-M-129*; *CTX-M-14*; *CTX-M-15*; *CTX-M-2*; *CTX-M-55*; *CTX-M-65*; *DHA-6*; *IMP-1*; *IMP-4*; *KLUG-1*; *KPC-2*; *KPC-3*; *mecI*; *metallo-beta-lactamase*; *NDM-4*; *NDM-5*; *OXA-1*; *OXA-10*; *OXA-181*; *OXA-2*; *OXA-29*; *OXA-48*; *OXA-9*; *PSE-1*; *SHV*; *SHV-1*; *SHV-5*; *TEM-1*; *TEM-135*; *TEM-2*; *VEB-1*; *VEB-3*
Chloramphenicol	*cat*; *catA*; *catB*; *cmlA*; *floR*
Colistin	*MCR-1*
MLS	*ermB*; *ermC*; *mphA*
Quinolone	*qnrA*; *qnrB*; *qnrS*
Rifamycin	*arr*
Sulfonamide	*sul1*; *sul2*; *sul3*
Tetracycline	*tetA*; *tetB*; *tetC*; *tetD*; *tetG*; *tetM*; *tetX*
Trimethoprim	*dfrA1*; *dfrA12*; *dfrA14*; *dfrA17*; *dfrA23*; *dfrA5*

To examine ARG transfer across multiple plasmids, we constructed a network that connected 1624 plasmids and 7129 recently transferred ARGs (Fig. 2B). In the network, when an ARG is recently transferred between two plasmids, both plasmids are linked to this specific ARG by edges (Fig. 2B). Therefore, the density of the edges quantitatively represents the frequency that a specific ARG transfers across certain numbers of plasmids. From the network, we found that each recently transferred ARG subtype was able to transfer across at least three different plasmids. Approximately 41.3% of ARG subtypes were able to transfer across more than 100 plasmids, indicating the extensive transfer of a large number of ARGs across very diverse plasmids. We also quantified the frequency of 7129 recently transferred ARGs across 1624 plasmids and compared these frequencies with plasmid-borne ARG abundances. The transfer frequency of the recently transferred ARGs was positively correlated with the abundance of plasmid-harboring ARGs (Fig. 2C, Spearman’s *r* = 0.612, *P* < 2.2e-16, Supplementary Fig. S3), indicating the close relationship between plasmid-borne ARG abundance and the horizontal transfer of ARGs across plasmids. Transfer frequency of ARGs among clinical plasmids is significantly higher than that in environmental plasmids (Wilcoxon test, *P* = 3.2e-15, Supplementary Fig. S4A). Three major plasmid-borne ARG types including beta-lactams, aminoglycosides, and sulfonamide resistance genes exhibit a higher transfer frequency of ARGs in clinical plasmids (Supplementary Fig. S4B). Only tetracycline resistance genes exhibit a higher HGT frequency in environmental plasmids. Collectively, the large collective of complete plasmid sequence combined with bioinformatic analysis depict a global map of inter-plasmid ARG transfer.

Many plasmids also carry resistances to common nonantibiotic biocides and heavy metals, which have been shown to be able to trigger the emergence and transmission of ARGs even in the absence of antibiotic exposure. Therefore, we also evaluated MRGs transfer among these collecting plasmids. As shown in Supplementary Fig. S5, 34 MRGs were observed to transfer among clinically relevant plasmids and 39 MRGs transfer among environmental plasmids, suggesting extensive MRG transfer among plasmids. Among these transferable MRGs, resistance genes against copper, silver, arsenic, and mercury exhibit a much higher HGT frequency. A larger proportion of environmental plasmids (15.5%) is responsible for only MRG transfer, which was higher than that of clinically relevant plasmids (3.5%) (Supplementary Fig. S6A and B). This might be associated with ubiquitous occurrence of various metals in the environment. The inter-plasmid transfer of genes would facilitate the co-occurrence of ARGs and MRGs in plasmids. Around 17.1% of total plasmids carried transferable ARGs and MRGs in clinical plasmids, while this proportion is 6.7% in environmental plasmids (Supplementary Fig. S6A and B). Among plasmids carrying transferable ARGs and MRGs, it was found that MRGs resistant to copper, silver, arsenic, and mercury mainly co-occur with beta-lactam and aminoglycoside resistance (Supplementary Fig. S6C and D).

### Antibiotic resistance gene transmission primarily occurs between compatible plasmids

**Figure 3:**
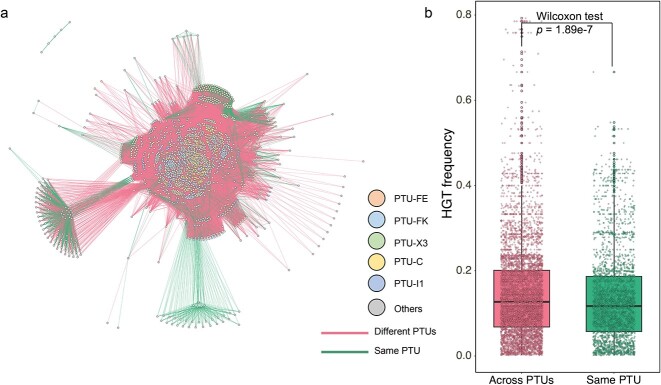
Horizontal gene transfer events among different plasmid types; (A) ARG transfers among compatible or incompatible plasmids; node indicate plasmid taxonomic units; the edges indicate ARG transfers between two nodes; (B) ARG occurrences in compatible or incompatible plasmids.

A large number of plasmids were frequently involved in inter-plasmid ARG transfer, yet it was still unknown whether phylogenetic differences in plasmids would hinder or facilitate the ARG recruitment. To test this, we further quantified ARG transfer across compatible or incompatible plasmids. Compatible plasmids represent the ability of two or more plasmids to coexist stably over a number of generations in the same bacterial cell line, while incompatible plasmids show inability to coexist in the same bacterial host [16]. Results demonstrated that ARGs preferentially transfer with a higher frequency across compatible plasmids compared to incompatible plasmids. Among 2420 clinical plasmid collections, 87.9% of the total ARG transfer events occurred between compatible plasmids (Fig. 3A), with only 12.1% occurring among incompatible plasmids. Moreover, ARG transfer frequency between compatible plasmids (average of 0.15) was significantly higher than that between incompatible plasmids (average of 0.14) (Fig. 3B, Wilcoxon test, *P* < .001), suggesting that the inter-plasmid ARG transfer is inclined to occur intracellularly. These inter-plasmid ARG transfer events mainly occurred in plasmid taxonomic units PTU-FE, PTU-FK, PTU-X3, PTU-C, PTU-I1, PTU-N1, and PTU-L/M, accounting for 67.5% of ARG transfer events.

### Insertion sequences and integrons contribute to the spread of antibiotic resistance genes among plasmids

**Figure 4:**
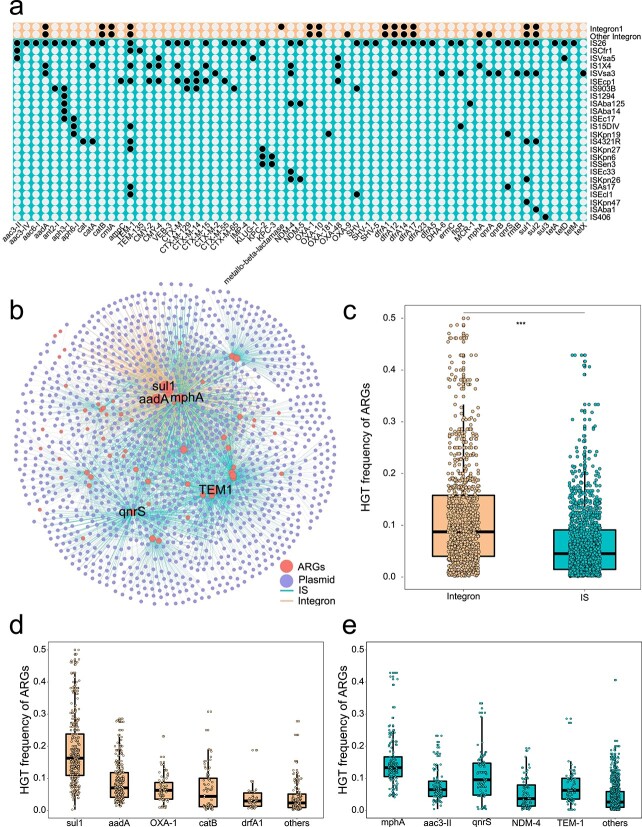
HGT frequency of recent transferred ARGs mediated by MGEs; (A) prevalence of ARGs mediated by mobile genetic elements, the dots indicate a specific ARG subtype was mediated by MGEs; (B) network analysis illustrates ARG transfer mediated by MGEs (IS and integrons); the dots represent ARG or plasmids; the ARG connecting two plasmids indicated that ARG transfer occurred in two plasmids; the edges depict the MGE-mediated transfer; (C) a boxplot is plotted showing frequency of ARG transfer mediated by MGEs; frequency of top five ARG transfer mediated by (D) integron and (E) ISs.

To determine whether MGEs were associated with recently transferred ARGs, we scanned sequences adjacent to recently transferred ARGs in plasmids. Results showed that both integrons and ISs exhibit a critical role in ARG transfer across compatible and incompatible plasmids. Among MGEs associated with recently transferred ARGs, a total of 24 IS families were identified. Specifically, IS*26* accounted for 63.1% of total ISs, following by IS*As17* (13.55%) and IS*Ecp1* (7.14%) (Fig. 4A). Apart from the IS families, integrons including Class 1 and Class 2 were also closely associated with recently transferred ARGs. Quantitative comparisons suggested that the IS families play a more significant role in mediating these recently transferred ARGs, compared to integrons. For example, a total of 56 ARG were associated with ISs, while only 16 ARG subtypes linked with integrons (Fig. 4A and B).

To further evaluate the role of ISs and integrons in the spread of ARGs across plasmids, ARG transfer frequency mediated by integrons or ISs was calculated. Results showed that integron-mediated ARG transfer frequency was significantly higher than that mediated by ISs (Wilcoxon test, *P* < .001, Fig. 4C). Among integron-associated ARGs, *sul1* exhibited the highest transfer frequency, followed by *aadA*, *bla_OXA-1_*, *catB*, and *dfrA1* (Fig. 4D). The gene *sul1* is often physically linked to Class 1 integrons, so the high transfer frequency of *sul1* could be associated with whole integron transfer. As for IS-associated ARGs, *mphA* showed the highest transfer frequency, followed by *aac(3″)-II*, *qnrS*, *bla_NDM-4_*, and *bla_TEM-1_* (Fig. 4E).

### Experiments validate IS*26*-mediated inter-plasmid antibiotic resistance gene transfer

To confirm the proposed mechanism of IS*26* mediating the inter-plasmid ARG transfer, we experimentally reproduced the transfer pathway of a gentamicin resistance gene *aac3-Ia* (*Gm^R^*) and tracked its transfer behavior across plasmids. The gene cassette of IS*26*-P*lac*-gfp_mut3b_-Gm^+^-IS*26* was cloned into the EcoRI and HindIII site of the pUC57 plasmid, and a *lac*I^q^ repressible promoter was cloned into the upstream of *gfp*_mut3b_ of the pUC57 plasmid, encoding for the GFP. As a result, there is no gfp expression in the strains harboring plasmid pUC57-*lac*I^q^-IS*26*-P*lac*-gfp_mut3b_-Gm^+^-IS*26*, but upon IS*26* transfer to a plasmid, gfp expression results in green fluorescent cells.

**Figure 5:**
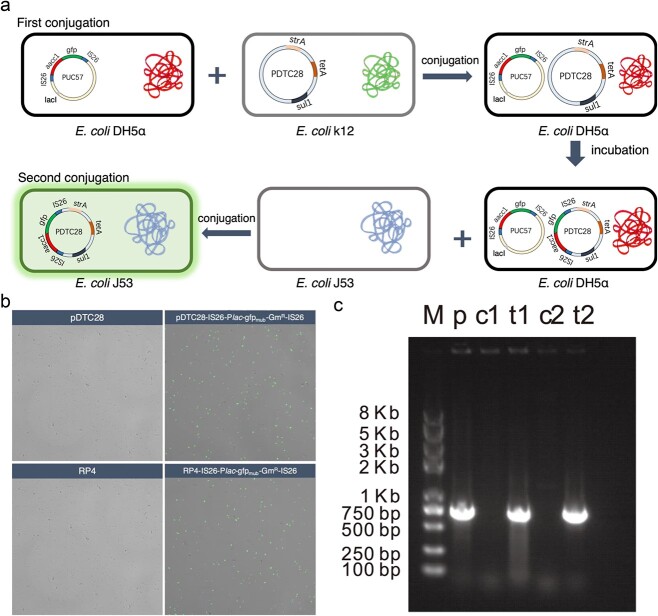
Experimental validation IS26-mediated inter-plasmid ARG transfer; (A) experimental design; (B) confocal laser scanning microscopy of IS26-mediating ARG transfer between plasmids; (C) gel electrophoresis of *aacC1* gene in the pTDC28 and RP4; M: DNA ladder; p: positive control (pUC57-*lacI^q^*-IS26-P*lac*-*gfp_mut3b_*-Gm^+^-IS26); c1: pDTC28; t1: pDTC28-IS26-P*lac*-*gfp_mut3b_*-Gm^+^-IS26; c2: RP4; t2: RP4- IS26-P*lac*-*gfp_mut3b_*-Gm^+^-IS26; the full length of aacC1 is 813 bp.

We used *E. coli* DH5α that contains the plasmid pUC57 carrying the IS*26*-associated Gm^+^ (pUC57-*lacI^q^*-IS*26*-P*lac*-*gfp_mut3b_*-Gm^+^-IS*26*) as the original source of the IS gene. Then, the pDTC28 was conjugatively introduced into *E.coli* DH5α, and the cointegrates formed between two plasmids were selected using LB-selective plates containing multiple antibiotics (ampicillin, tetracycline, and gentamicin). Considering *lac*I^q^ suppresses the expression of gfp, the fluorescent protein expression is suppressed even if IS*26*-mediated transfer occurred from pUC57 to pDTC28 within the same bacterial host. Therefore, a second conjugation transfer assay was conducted to identify any IS*26*-mediated transfer. Cointegrates harboring pUC57 and pDTC28 were selected as the donor, while *E.coli* J53 without plasmid was the recipient. After mating, the transconjugant *E.coli* J53 carrying pDTC28 was selected, and the fluorescent expression could be identified if the IS*26* transfer event occurred (Fig. 5A). After 12-h incubation of cointegrates of pUC57 and pDTC28, fluorescent expression was observed in *E.coli* J53, evidenced by the CLSM image (Fig. 5B). Colonies were also tested for the transfer of IS gene by PCR (Fig. 5C). These results collectively confirmed IS*26* transfers across plasmids. To demonstrate the ubiquitous IS*26*-mediated transfer across plasmids, the RP4 plasmid was also selected as the recipient for the IS gene. Finally, we found that IS*26* transfer events also occurred from the plasmid pUC57 to RP4, according to both CLSM (Fig. 5B) and PCR results (Fig. 5C).

### Inter-plasmid antibiotic resistance gene transfers facilitate antibiotic resistance propagation in clinical pathogens

**Figure 6:**
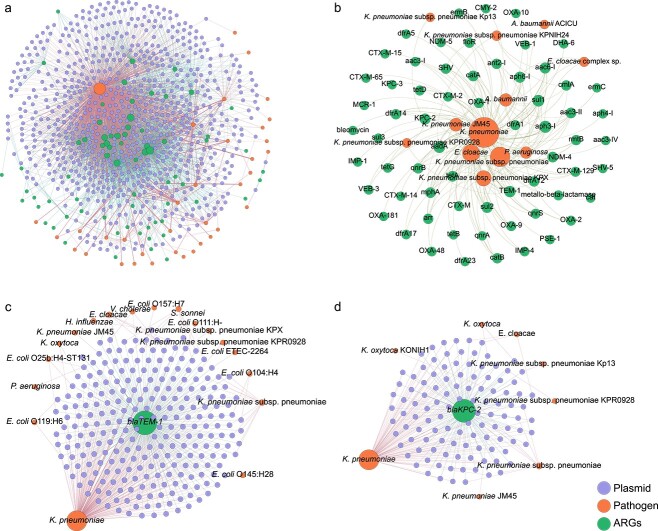
Transfer of ARGs among plasmids that carried by pathogens; (A) network illustrated ARG transfer among plasmid carried by pathogens; the dots represent ARGs, plasmids, and pathogens; the edges connecting ARGs and plasmids depict that ARG transfer among plasmids, and edge connecting plasmid and pathogens showed plasmids carried by pathogens; (D) network showed ARGs transfer among ESKAPE pathogens; representative ARGs of (C) *bla_TEM-1_* and (D) *bla_KPC-2_* transfer among plasmid that carried by pathogens.

To investigate how the dissemination of ARGs across plasmids contributes to the emergence of antibiotic resistance in pathogenic bacteria, we constructed a network to link inter-plasmid ARG transfer and antibiotic resistance in various pathogens. We first generated a multilevel network consisting of 3191 ARG transfers across 673 pathogen-harboring plasmids. These inter-plasmid ARG transfers facilitate antibiotic resistance propagation in 36 pathogens (Fig. 6A), including *K. pneumoniae*, *E. coli*, *A. baumannii*, and *P. aeruginosa*. Among these pathogens, 90.3% were categorized as ESKAPE (*E. faecium*, *S. aureus*, *K. pneumoniae*, *A. baumannii*, *P. aeruginosa*, and *Enterobacter* sp.) pathogens. As shown in Fig. 6B, 64 ARG subtypes were observed to be transferred across ESKAPE pathogens. Noticeable, several ARGs against last line antibiotics, e.g. *bla_NDM_* and *bla_KPC_* genes against carbapenemase, could transfer across plasmids harbored by ESKAPE pathogens. This highlights the critical role of plasmids in recruiting ARGs and in contributing to antimicrobial resistance in ESKAPE pathogens.

To further depict which ARGs are transferred across these pathogens, four representative beta-lactam resistance genes (*bla_TEM-1_*, *bla_NDM-4_*, *bla_KPC-2_*, and *bla_SHV-1_*) were selected to construct a multilevel network. Results demonstrated that development of antibiotic resistance in diverse pathogens has been expedited by frequent ARG movement. As shown in Fig. 6 and Supplementary Fig. S7, inter-plasmid ARG transfer networks contained two layers, including the inner layer connecting recently transferred ARGs with plasmids, while the outer layer linked plasmids with pathogens. Interestingly, the gene *bla_TEM-1_*, the ARG encoding resistance to beta-lactams [35], was observed to be connected with the most variety of 550 clinically relevant plasmids and endowed antibiotic resistance to the most diversity of 18 clinical pathogens, e.g. *K. pneumoniae*, *E. coli* ETEC, and *P. aeruginosa* (Fig. 6C). Similarly, other beta-lactam resistance genes (*bla_KPC-2_*, *bla_NDM-4_*, and *bla_SHV-1_*) were also observed to transfer across multiple plasmids, conferring carbapenem and extended-spectrum beta-lactamase resistance determinants to a variety of clinical pathogens (Fig. 6D, Supplementary Fig. S7A and B). To be specific, *bla_KPC-2_* and *bla_NDM-4_* were observed to have transferred across eight pathogens and *bla_SHV-1_* transferred among five pathogens. These results demonstrate that extensive ARG transfer across plasmid-harboring pathogens is a critical mechanism for pathogens to adapt to widely used antibiotics in clinical settings.

### Inter-plasmid antibiotic resistance genes transfer across the ecological boundary of clinical and environmental settings

**Figure 7:**
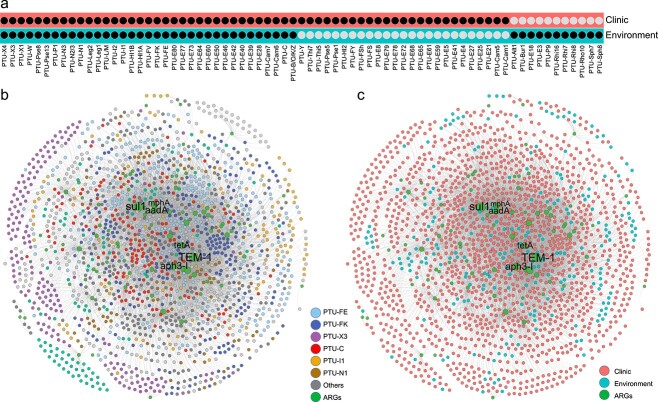
Transfer of ARGs in plasmids across clinical and environmental strains; (A) plasmid taxonomic units identified in clinical and environmental plasmids; (B) network of ARG in all plasmids; the nodes indicate plasmid taxonomic units and ARGs; the edges present ARG transfer events between two plasmids; (C) network reveals ARG transfer among plasmids isolated from clinic and environment; the nodes indicate clinical plasmids, environmental plasmids, and ARGs; the edges represent ARG transfer events between clinical and environmental plasmids.

Pathogenic bacteria and clinically relevant ARGs are discharged via wastewater treatment plants into receiving water environments, and these ARGs readily become part of the environmental resistome [36-38]. The maintenance, proliferation, and fate of these ARGs have received growing attention, with the potential for eventual transfer back to various pathogens that cause serious human infections [39, 40]. In recent years, a variety of clinically relevant plasmids has been isolated from environmental compartments. However, it is still unknown whether clinical plasmid-borne ARGs could transfer resistance determinants to environmental plasmids. Therefore, it is important to investigate whether those ARGs transcend clinical and environmental boundaries. We further investigated the inter-plasmid ARG transfer across clinical and environmental settings. Results suggest that ARGs are transferred beyond the clinical boundaries (Fig. 7). Our collection of 2420 clinically relevant plasmids are categorized as 60 plasmid taxonomic units, among which 35 groups are shared with the environmental category, including PTU-FE, PTU-FK, PTU-X3, PTU-C, and PTU-I1 (Fig. 7A). These shared plasmid types represent frequent communication between plasmids across clinical and environmental settings.

A total of 7696 recently transferred ARGs were identified from clinical and environmental plasmids, encoding resistance to beta-lactams, aminoglycosides, sulfonamide, MLS, quinolone, and chloramphenicol (Fig. 7B and C). Among these recently transferred ARGs, 76.5% of ARGs were observed to transfer across shared plasmid types, suggesting that plasmid transmission plays an important role in the inter-plasmid ARG transfer. Importantly, some ARGs encoding resistance to last-line antibiotics, such as *bla_NDM_*, *bla_CTX-M_*, *bla_KPC_*, *bla_SHV_*, and *mcr-1*, were also transferrable across clinical and environmental settings. These inter-plasmid ARG transfer events across ecological boundaries might explain their rapid dissemination. Collectively, these results provide evidence for the inter-plasmid ARG transfer across ecological boundaries.

## Discussion

### A global map of inter-plasmid antibiotic resistance gene transfer

Plasmids can shuttle ARGs across phylogenetically distant bacteria, thus broadening the bacterial host range of antibiotic resistance. Recently, the emergence of multidrug-resistant plasmids has been increasingly identified in clinical settings, and each of these plasmids individually carries a different linkage of multidrug resistance genes [41-43]. Therefore, it is necessary to establish a global map of inter-plasmid ARG movement to advance our understanding about when and how ARG movements occur on these plasmids, thus facilitating the development of control strategies to combat the rise of multiple resistances in pathogens. Through systematically bioinformatic examination of 3302 plasmids, this study provided a global map of inter-plasmid ARG transfer via either compatible or incompatible plasmids, in which 2420 whole-genome sequenced plasmids with 7129 potential ARG transfers across 36 pathogens were observed. Based on the global map, a universal pathway of extensive inter-plasmid ARG transfer was revealed to show genetic linkage of multiple resistance genes on the same plasmid. More than 73% of total antibiotic resistance plasmids were observed to carry more than two ARGs in our collected plasmids. Interestingly, the ARG transfer proportion between compatible plasmids is four times higher than that in incompatible plasmids. This is partly attributed to the major contribution of coexistence of plasmids in the same bacterial host, as it provides more opportunities for ARG transfer via plasmid fusion or homologous recombination [44, 45]. It was estimated that almost 50% bacteria carried more than one plasmid [46]. The compatible plasmids coresiding in the same bacterial host provide an ecological niche for plasmids to exchange ARGs. This might be a general adaptation strategy for bacteria in response to various environmental stresses, thus enabling the high ARG-transfer frequency. Collectively, our integrated bioinformatic pipeline was established to obtain a global map of ARG movement between plasmids.

The established bioinformatic pipeline is an effective and robust approach to identity horizontal gene transfer between different bacterial species, which has been applied to explore gene exchange in human microbiomes [27, 28, 30], as well as ARG transfer across bacterial species [18, 26, 29]. These previous publications also used publicly available database to conduct qualitative or quantitative evaluation of horizontal gene transfer. It should be noted that plasmid conjugative transfer could also cause the nearly identical ARG nucleotide sequence among different bacterial species. To exclude the effect of plasmid conjugative transfer on observed recently transferred ARGs, plasmid pairwise alignment was conducted and stringent cut-values (nucleotide identity <80% and coverage <80%) were used to retain the phylogenetically distant plasmid between different bacterial species. These stringent cut-values enable to ensure that observed recently transferred ARGs are attributed to inter-plasmid ARG transfer events.

### ISs and integrons are the main drivers for plasmid to recruit antibiotic resistance genes

ISs and integrons are MGEs involved in genome rearrangement, responsible for mobilization of ARGs between plasmid and chromosome, as well as between chromosomes. Previous studies also reported that ISs contribute to ARG transfer in sporadic fusion cases of specific plasmids, e.g. plasmids IncX [47], IncA/C [48], IncI [48], and IncF [49]. However, apart from these minority of plasmids, it remains unknown whether it is universal for ISs and integrons to contribute the inter-plasmid ARGs transfer. Our bioinformatic analysis in conjunction with *in vitro* experiments confirm that ISs and integrons are the main drivers for plasmids to acquire ARGs from compatible plasmids. Through *in silico* genomic analysis of 2420 complete plasmid genomes, we identified that a total of 22 IS elements facilitated the extensive transfer of antibiotic resistance genes (ARGs) across 1250 plasmids, representing 33 plasmid taxonomic units in 36 pathogens. This suggested that the ISs mediate the inter-plasmid ARG transfer is a universal mechanism for plasmid to recruit ARGs. In particular, IS*26* mediated the most abundant ARG movements during the inter-plasmid transfer process, which was further validated by the *in vitro* experiment. Notably, the transposition mediated by IS*26* rapidly occurred within 24 h, highlighting the highly dynamic nature of IS*26* in the plasmid evolution process. This highlights the significant role of IS*26* in ARG dissemination, especially given that IS*26* is closely associated with pathogen-borne plasmids, such as IncFIA [50], IncFIB [50], IncI1 [51], and IncX4 [52]. IS*1294* was also reported to mediate *bla_CMY-2_* transfer between a pair of compatible plasmids of IncI and IncA/C [48]. In addition to compatible plasmids, we also found that IS*26* could mediate ARG transfer between incompatible plasmids. Incompatible plasmids generally do not coexist within the same bacterial cell, thus limiting direct ARG exchange. In this case, the bacterial chromosome may serve as a “staging post” facilitating genetic exchange via homologous recombination between plasmids and chromosomes, as documented in previous studies [18]. It is assumed that ARG transfers across incompatible plasmids might occur through an indirect process, in which plasmid and chromosomal recombination first occurs within one bacterial host, exchanging ARGs between chromosomes and plasmids. Further studies are required to explore this potential mechanism of ARG exchange between incompatible plasmids. ISs associated with ARGs might be underestimated because some “resistance transposons” (e.g. Tn*916*, Tn*925*, and Tn*1545*) larger than 10 kb [53] would be filtered out in our study. Additionally, ISs would have been lost over time, leading to the stabilization of ARGs in a diverse range of plasmid backgrounds [9], e.g. ISApl*1*, which might also have underestimated ISs associated with ARGs.

### Multiple selection pressures facilitate antibiotic resistance gene movement across plasmids

Antibiotics usually serve as selection pressures to promote ARG movement across plasmids. Antibiotic concentrations in the environment are relatively low (e.g. aquatic environments, 10 ng/l–10 μg/l) [54], which are much lower than therapeutic dosages in human blood plasma (e.g. ampicillin range 5–230 mg/L) [55]. Thus, the inter-plasmid ARG transfer occurred more frequently in clinical settings than that in the environment settings (Supplementary Fig. S4A), indicating that the intensive use of antibiotics in clinics might pose a stronger driving force to facilitate ARG movement across these plasmids. Due to the intensive use of antibiotics, hospitals have been recognized as a hotspot for IS-mediated ARG rearrangement across diverse genetic contexts. In hospital settings, multiple plasmids were observed to be involved in IS-mediated ARG transfer events, e.g. IS*26*-mediated *bla_KPC-2_* [50], IS*6*-mediated *bla_IMP-8_* [41], encoding resistance to carbapenems. The driving force of complex antibiotics not only promotes frequent ARG transfer across plasmids but also subsequently facilitates the generation of multidrug-resistant plasmids via genetic linkage of multiple ARGs. These multidrug-resistant plasmids can be selected and persist in pathogens under antibiotics stresses. The transfer of beta-lactam and aminoglycoside resistance genes between clinical multidrug-resistant plasmids is much more frequent than that in the environment, partly because beta-lactams and aminoglycosides are commonly used antibiotics in clinical settings [56]. In contrast, tetracycline resistance gene transfer frequency was higher in environmental plasmids.

Previous studies have documented the co-occurrence of multiple antibiotic resistance genes (ARGs) and metal resistance genes (MRGs) in IncP plasmids [57, 58]. A recent study based on 4582 plasmid collection revealed that nearly 5% of plasmids harbor both ARGs and MRGs in plasmids genomes [59]. However, it remains unknown about why the ARGs were genetically linked with MRGs within same plasmid genome. In this study, we demonstrate that MRGs could also hop across multiple plasmids. Approximately 17.1% of clinical plasmids and 6.7% of environmental plasmids were observed to carry both ARGs and MRGs. Due to the genetic linkage of ARGs and MRGs in plasmids [57-60], heavy metals act as a co-selective agent in the emergence and spread of antibiotic resistance [61]. In addition, the proportion of MRGs transfer across environmental plasmids is much higher (15.5%) than that of clinical plasmids (3.5%), which might be associated with higher heavy metal contaminations in the environment compared to clinic settings [62, 63]. Unlike antibiotics, metals are not subject to degradation and can subsequently represent a long-standing pressure to maintain a pool of ARGs and MRGs [62]. Previous studies also revealed that nonantibiotic pharmaceuticals could contribute to the transmission of ARGs via plasmid conjugation or transformation [64, 65], whereas their influence on epidemiology needs further exploration.

### Inter-plasmid antibiotic resistance gene transfer explained the rapid increase of antibiotic resistance in ESPAKE pathogens

The World Health Organization (WHO) has listed ESKAPE pathogens for which new antibiotics are urgently needed, including multidrug-resistant *Enterococcus* [66]; vancomycin-resistant and methicillin-resistant *S. aureus* [67], *K. pneumoniae* [68], *A. baumannii*, and *P. aeruginosa* [69]; and *Enterobacter* spp. [13]. The acquisition of ARGs by ESKAPE pathogens has posed a great challenge to clinical infection treatments, increasing mortality and the burden of disease [70]. Our study provides a synoptic view of the ARG exchange network in ESKAPE pathogens, such as the most worrying *mcr-1* against polymyxin, *bla*_*NDM-5*,_ and *bla_KPC2_* against carbapenems antibiotics. The WHO reported that global antibiotic resistance in bacteriological infections caused by *K. pneumoniae* was increased from 2016 to 2020, including third-generation cephalosporins, carbapenems, and fluoroquinolones [71]. Our finding of inter-plasmid ARG transfer provides the explanation for global increase of antibiotic resistance in *K. pneumoniae*. In this study, *K. pneumoniae* is a reservoir for the inter-plasmid ARG transfer, given that it contributes 85.5% of all recently transferred ARGs in the clinically relevant plasmids in this network. From the multilevel network, 3035 ARGs were observed to transfer across 606 plasmids harbored by *K. pneumoniae*, thereby increasing resistance of *K. pneumoniae* against clinical antibiotics, including aminoglycoside, third-generation cephalosporins, and carbapenems. Compared to *K. pneumoniae*, other ESKAPE pathogens, e.g. *Enterobacter* spp., *P. aeruginosa*, and *A. baumannii*, also participate in the inter-plasmid ARG transfer, but with relatively lower frequencies (2.2%, 1.6%, and 1.1%, respectively).

As ARGs can spread across species boundaries, it is critical to understand the dissemination routes that connect humans and environmental microbiota. ARGs in the environment can transmit to human pathogenic bacteria and eventually cause untreatable infections. Our study found that the clinical plasmids in *K. pneumoniae* can acquire diverse ARGs, in which 59.5% of ARGs derived from environmental plasmids could be captured by *K. pneumoniae* carrying plasmids, e.g. *bla_NDM-4_*, *bla_KPC-2_*, and *mcr-1*. In addition to *K. pneumoniae*, other pathogens (e.g. *A. baumannii*, *P. aeruginosa*, and *Enterobacter* spp.) also exchange ARGs with environmentally derived plasmids, but with a relatively lower transfer frequency (17.5%, 15.9%, and 5.2% of total environmentally derived ARGs). Environmentally derived plasmids could disseminate to clinical settings, as evidenced by a case study where a patient was infected by bacteria-harboring *bla_NDM-1_* on a plasmid originating from drinking water or seepage samples in New Delhi [39]. Once an environment-derived plasmid is acquired by a clinical pathogen, it would then allow a rapid inter-plasmid ARG transfer. Thus, the inter-plasmid exchange of ARGs across environmental and clinical boundaries could expand the resistance spectrum of pathogens.

In summary, inter-plasmid ARG movement was investigated from phylogenetically distant plasmid in both clinical and environmental settings by integrating powerful genomic analysis with a large fully sequenced plasmids collection. A universal pathway of inter-plasmid ARG transfer among clinical pathogens was proposed to explain the rapid emergence and dissemination of antimicrobial resistance in ESKAPE pathogens. Not only for intra-ecosystem, the inter-plasmid ARG transfer is also happening across clinical and environmental ecosystem boundaries, thus raising a concern of the environmental resistome serving as a reservoir that facilitates ARG maintenance and transmission back to clinical pathogens. These findings advance our understanding of the emergence of multidrug-resistant plasmids and ARG propagation in commensal and pathogenic bacteria, thus facilitating to develop effective strategies to mitigate the increasing trend of antimicrobial resistance globally.

## Author contributions

Yi Luo, Daqing Mao, Jianhua Guo, and Xiaolong Wang conceived and designed this study. Xiaolong Wang collected data and performed the bioinformatic analysis, data analysis, and inter-plasmid ARG transfer experiment. Xiaolong Wang and Hanhui Zhang performed the visualization of all data and the artistic design of all figures. Shenbo Yu and Donghang Li helped to construct the plasmids. Yi Luo, Daqing Mao, Jianhua Guo, and Xiaolong Wang provided critical interpretations of the data. Yi Luo, Daqing Mao, and Xiaolong Wang wrote the manuscript. Yi Luo, Daqing Mao, Jianhua Guo, Michael R. Gillings, and Hongqiang Ren contributed substantially to revisions.

## Conflicts of interest

None declared.

## Funding

This work was supported by the Key Projects of the National Natural Science Foundation of China (41831287), the National Key R&D Program of China (2020YFC1806904), and by the National Natural Science Foundation of China (31870351, 42107456).

## Data availability

Plasmid genomes used in this study were downloaded from the NCBI RefSeq database (ftp://ftp.ncbi.nlm.nih.gov/refseq/release/plasmid/).

## Supplementary Material

Table_S1_Full_information_of_clinic_category_plasmids_wrad032

Table_S2_wrad032

Table_S3_wrad032

Table_S4_wrad032

Table_S5_wrad032

Table_S6_wrad032
